# Chinese Medicine Formula “Jian-Pi-Zhi-Dong Decoction” Attenuates Tourette Syndrome via Downregulating the Expression of Dopamine Transporter in Mice

**DOI:** 10.1155/2013/385685

**Published:** 2013-02-03

**Authors:** Dao-han Wang, Wei Li, Xiao-fang Liu, Jin-ming Zhang, Su-mei Wang

**Affiliations:** ^1^Department of Pediatrics, Dongfang Hospital, Beijing University of Traditional Chinese Medicine, Beijing 100078, China; ^2^Department of Nuclear Medicine, Chinese PLA General Hospital, Beijing 100853, China

## Abstract

Jian-Pi-Zhi-Dong Decoction (JPZDD) is dedicated to the treatment for Tourette syndrome (TS) with the guidance of the theories of Traditional Chinese Medicine (TCM). This study aims to investigate the expression of dopamine transporter (DAT) in the striatum and stereotyped behavior of TS mice model by intervention of JPZDD. Mice were induced by 3,3′-iminodipropionitrile (IDPN, 350 mg kg^−1^ day^−1^, i.p.) for 7 days and divided into 4 groups (*n* = 20, each): control and IDPN groups were gavaged with saline and the remaining 2 groups with Tiapride (Tia, 50 mg kg^−1^ day^−1^) and JPZDD (20 g kg^−1^ day^−1^), respectively. The results showed that the scores of stereotyped behavior in IDPN+JPZDD group were significantly reduced. A noticeably increased ^11^C-**β**-CFT binding at bilateral striatum was observed after administration of JPZDD versus that of IDPN or Tia. Immunohistochemistry and in situ hybridization studies manifested higher levels of DAT protein and mRNA in IDPN+JPZDD group. These findings not only demonstrated that JPZDD could effectively inhibit the abnormal behaviors of TS mice model, but also increase the level of DAT in striatum. Therefore, JPZDD could be one of potential treatments of patients with TS.

## 1. Introduction

Tourette syndrome (TS) is a chronic neurobehavioral disorder characterized by involuntary motor and phonic tics. TS usually starts at childhood with peak age from 7 to 15 years and persists to late adolescence or even early adulthood. Tics are more common in males than in females with the ratio of 3–9 : 1 [1]. The morbidity in children is about 1% while only 0.3%–0.5% in adults. 35%–50% TS children could trace back to their affected first-degree relatives [2, 3]. In addition, TS patients often accompanied with attention deficit hyperactivity disorder (ADHD) and obsessive compulsive disorder (OCD).

Abnormal dopaminergic activity in the synaptic cleft has been considered as an important factor in TS for a long time. This hypothesis is supported by several evidences such as excessive dopaminergic activity in the nigrostriatum of basal ganglia circuit, releasing of surplus DA during tonic phase and the beneficial effect of tentative medication of DA precursor levodopa (L-DOPA) or DA antagonists on clinic symptoms [4]. Dopamine transporter (DAT), a glycoprotein localized exclusively at presynaptic membrane of DA neurons, is responsible for modulating the concentration of extrasynaptic DA by reuptaking it into neurons and therefore plays an important role in keeping the phasic-tonic balance of DA releasing. A Functional neuroimaging study suggested that reduced activity of DAT in regulation of tonic DA releasing might underlie subsequent pathological states in TS [5]. 

It has been documented that psychomotor stimulant drugs like amphetamine and cocaine may be the causes of tics from clinical pharmacology. Moreover, methamphetamine at different doses could increase locomotor activity or elicit stereotypic behavior in rats [6, 7]. These excitatory substances act as substrates to block DAT-mediated uptake of DA or reversely promote DAT-mediated DA efflux [8].

Previous clinical study has proven that Jian-Pi-Zhi-Dong Decoction (JPZDD) was an effective antitics agent with overall response rate of reducing tics at 86.8% [9]. The present study aimed to evaluate the anti-tics function of JPZDD from an animal model, and explored tentatively if abnormal expression of DAT existing in TS has relevance to severity of stereotyped behavior.

## 2. Materials and Methods

### 2.1. Drugs and Reagents

3,3′-iminodipropionitrile (IDPN) was purchase from Sigma-Aldrich Co. LLC. (USA), Tiapride (Tia) from Jiangsu Nhwa Pharmaceutical Co. Ltd. (Jiangsu, China), rat anti-N-terminal of DAT monoclonal antibody (primary antibody, 1 : 1000) from Millipore Co. (Billerica, MA, USA), goat-anti-rat IgG (H+L) antibody (secondary antibody, 1 : 250) and rabbit ABC detection 1/4 kit from Vector Labs, Inc. (Burlingame, CA, USA), DAT mRNA in situ hybridization kit from Wuhan Boster Oio-Engineering Co. Ltd. (Wuhan, China), and ^11^C-*β*-CFT was synthesized and provided from Department of Nuclear Medicine, Chinese PLA General Hospital (Beijing, China).

### 2.2. Preparation of JPZDD

The JPZDD formula includes 10 different Chinese medicinal herbs (Table 1). They were purchased from pharmaceutical department of Dongfang Hospital affiliated to Beijing University of Chinese Medicine (BUCM). Director Qing-chun Hao identified components, and the voucher specimens were deposited. All of them were soaked for 1 h at room temperature and decocted with distilled water for 2 h. The filtrates were condensed and dried by a vacuum-desiccator at 60°C. The extracted granules were analyzed by infrared fingerprint compared with standards to guarantee the qualified rate of more than 90% then packaged and stored at room temperature.

### 2.3. Experimental Animals and Behavior Recordings

Eighty ICR mice (male, aged 4 weeks, and 18 ± 2 g) were purchased from Vital River Laboratories (Beijing, China). All animal experimental protocols conformed to the Animal Management Rules of the Chinese Ministry of Health, and the study was approved by the animal ethics committee of the Chinese Academy of Medical Sciences. They were housed 10 per cage in an air-conditioned animal room with 12 h light/dark cycle, allowed access to water and food ad libitum, maintained in a constant temperature 20 ± 2°C and humidity 50 ± 5%, and fed for 1 w before generating TS model. After 1 w, mice were randomly divided into the saline group (control group) (*n* = 20) and the TS model group (*n* = 60). The former were intraperitoneally injected (i.p.) with saline (0.9%) (15 mL kg^−1^); the later were injected with IDPN (350 mg kg^−1^, i.p.) once a day for 7 consecutive days. IDPN mice model groups were further divided into 3 groups: IDPN group (*n* = 20), IDPN+Tia group (*n* = 20), and IDPN+JPZDD group (*n* = 20). The ethological score between each group was balanced referring to the evaluating grade of stereotypy (Table 2) [10, 11]. The saline and IDPN groups were gavaged with saline (0.9%) at 20 mL kg^−1^, IDPN+Tia group with Tia at 50 mg kg^−1^, and IDPN+JPZDD group with JD at 20 g kg^−1^, respectively, once a day for 8 consecutive weeks. Behavioral recordings began once every 7 d after IPDN induction and drug administrations and were conducted by 2 trained observers, who were familiar to the measurements but blind to the group condition. Each animal was observed for 1 min of every 5 min for a total of 6 periods. One or more episodes which were in accordance with the grades got the corresponding score and calculated the average score on the basis of results from 2 observers, as the objective indicator of behavioral changes.

### 2.4. MicroPET Scans

Imaging study with ^11^C-*β*-CFT was conducted 1 d after the last behavior test of each mouse. ^11^C-*β*-CFT was synthesized from its corresponding precursors ^11^C-Triflate-CH_3_ and nor-*β*-CFT, with a radiochemical purity of more than 95% [12]. Each mouse was injected with 1 mCi ^11^C-*β*-CFT through the caudal vein. After 20–25 min till the radiotracer was generalized uptake, each was anesthetized with 3.5% chloral hydrate through intraperitoneal injection and fixed in a self-made animal framework on which 2 mice lay head-to-head in a prone position. Each was scanned 15 min with its brain centered in the axial and transaxial fields of view (Explore VISTA Micro-PET/CT scanner from GE Healthcare Co., USA). Image data were sorted into 3D sinograms, followed by Fourier rebinning and 2D ordered subset expectation maximization reconstruction with 2 iterations and 50 subsets. The images pixel size was 0.385 × 0.385 × 0.385 mm. For semiquantitative evaluation, the regions of interest (ROI) (right/left striatum and cerebellum) were measured 3 times, and the ratio of striatum to cerebellum was counted with mean value of the measurements.

### 2.5. Immunohistochemistry

Expression of DAT protein was detected by immunohistochemistry one day after PET scan. Mice were perfused and fixed with 4% PFA after anesthetizing with 10% chloral hydrate. The global brains were stripped off and soaked in 4% PFA for 14 h. The fixed brains were dehydrated by 30% sucrose for 24 h and embedded in OCT medium after cryoprotection. Frozen brains were cut into 30 *μ*m coronal sections prelocated at caudate putamen by a refrigerated microtome (Shandon, GB). Sections were immersed into 3% hydrogen peroxide solution to inactivate the endogenous peroxidase and blocked by 10% goat serum at room temperature, incubated by the primary antibody at 4°C overnight, then by the secondary antibody at room temperature for 2 h, finally by avidin-biotin-peroxidase complex for 90 min. Sections were visualized by DAB for 5 min then mounted by neutral balsam. 6 visual fields were chosen randomly from bilateral colored striatum under an upright microscope at 100x magnification. Quantifications were performed by Image-pro plus 6.0 analysis system to calculate the optical density (OD) of each field. The values of DAT protein were chosen and counted from every three brain slices of each mouse with similar forms.

### 2.6. In Situ Hybrization

Brain sections were deparaffinizated by mixed solution of 30% hydrogen dioxide and absolute methanol (1 : 50) for 30 min at room temperature. Several drops of diluted pepsin solution were added to slices for proteolysis and exposure of mRNA at 37°C for 2 min and fixed by 1% paraformaldehyde (PH = 7.2–7.6). Prehybridization was performed at 42°C for 4 h with prepared DAT mRNA in situ hybridization kit and hybridization at 42°C overnight in calorstat (SPX-150C, Boxun Industrial Co. Ltd., Shanghai, China). All sections were washed, blocked, incubated with streptavidin biotin-peroxidase complex (SABC) and biotin-peroxidase at 37°C for 30 min, and finally visualized with DAB for 30 min before alcoholic dehydration and mounting. The slices were observed and microphotographed under the light microscope. The values of OD reflecting the expression of DAT mRNA were determined by Metamorph image processing analysis system from Diagnostic Tool Electrics Co., USA.

### 2.7. Statistical Analysis

Results were expressed as the mean ± SEM. Statistical differences between groups were determined by one-way analysis of variance (ANOVA). Student-Newman-Keuls (SNK) test was employed for the comparison of parameters if the analysis of ANOVA had significant difference. All analyses were performed by the SPSS 18.0 (SPSS Inc., Chicago, IL, USA), and *P* value < 0.05 was accepted as statistically significant.

## 3. Results

### 3.1. Behavior Study

**Figure 1:**
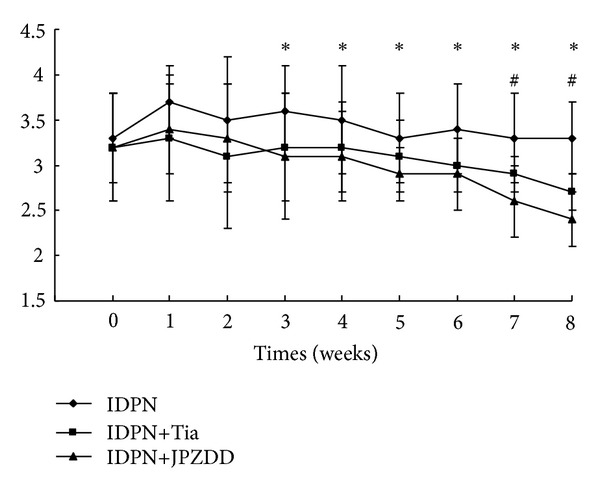
Evaluations of stereotypy of TS mice model in different groups during an 8-week period. The scores were recorded every 7 d. The initial scores before treatments showed no differences among groups (*P* > 0.05). However, there was a gradual increase in the scores in IDPN mice. Treatments of either Tia or JPZDD gradually decreased the stereotypy scores staring from 3 w of treatment. In addition, more significant reduction of stereotypy scores were observed in JPZDD treated mice. Data were indicated as the mean ± SEM (*n* = 20 mice/group); * indicates the *P* < 0.05 compared with IDPN group; ^#^ indicates the *P* < 0.05 compared with Tia group.

TS mouse model induced by IDPN showed abnormal stereotypes in different degrees. Remarkably, severity of stereotypes in either IDPN+Tia or IDPN+JPZDD group decreased significantly as compared to IDPN alone group (*P* < 0.05). Comparison between administrated groups showed the average score of JPZDD was lower than Tia (*P* < 0.05) at the end of experiment, although it was still higher than that of saline group (*P* < 0.05) (Figure 1).

### 3.2. MicroPET Imaging

**Figure 2:**
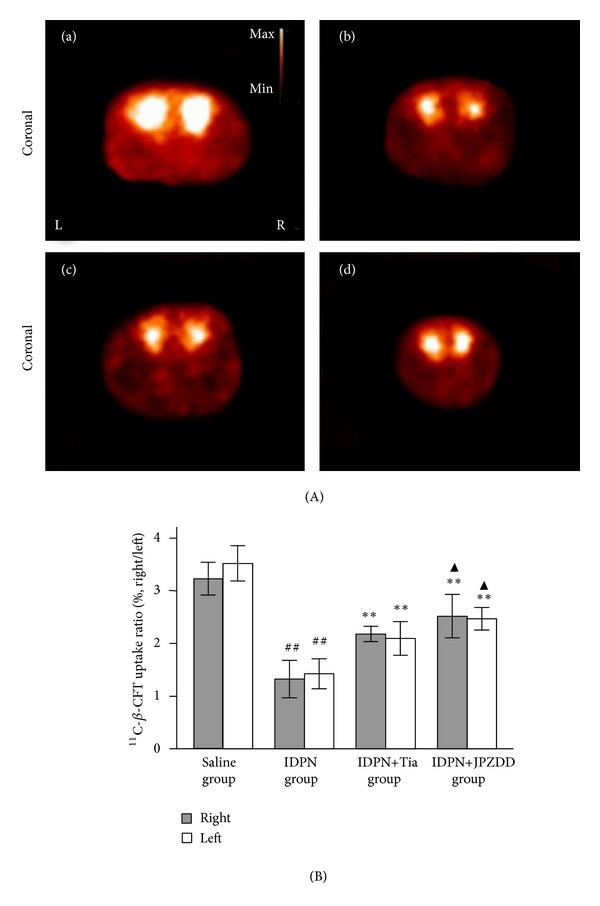
MicroPET imaging and semiquantitative determination of ROI: Panel (A) represents coronal MicroPET imaging of bilateral striatum regions after an 8-week treatment. The images were acquired after tail vein injection of 1 mCi ^11^C-*β*-CFT. (a) Saline group, (b) IDPN group, (c) IDPN+Tia group, and (d) IDPN+JPZDD group. Brighter areas revealed higher uptake ratio in brains. Panel (B) indicates ROI at bilateral striatum to cerebellum after ^11^C-*β*-CFT uptake and tracing. **P < 0.01 compared with IDPN group, ^##^ *P* < 0.01 compared with saline group, and ^▲^ *P* < 0.05 compared with IDPN+Tia group.

Coronal scans manifested by PET imaging showed high tracer accumulation at bilateral striatum regions in saline group with uniform distribution and symmetric morphous. Marked fuzzy images and low accumulation of ^11^C-*β*-CFT were observed in TS mice model (Figure 2(a)). After 8 w, the uptake ratio of ^11^C-*β*-CFT was significantly higher in both Tia and JPZDD treated groups than that in IDPN group (*P* < 0.01). Furthermore, the uptake ratio of ^11^C-*β*-CFT in IDPN+JPZDD group was significantly better than that in IDPN+Tia group (*P* < 0.05) (Figure 2(b)).

### 3.3. DAT Expressions in the Striatum

**Figure 3:**
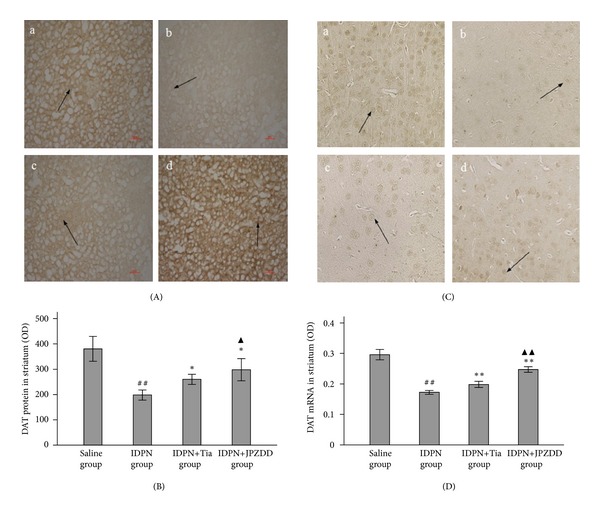
Immunostaining of DAT protein and in situ hybridization of DAT mRNA level in the striatum. Panel (A) displays the immunostaining of DAT protein (black arrow) in striatum in each group detected by immunohistochemistry. Magnification 100x. Panel (B) indicates the histogram of density of DAT protein. Data were represented as the mean ± SEM (*n* = 20 mice/group). Panel (C) displays the expressions of DAT mRNA (black arrow) in striatum from each group detected by in situ hybrization. Magnification 40x. Panel (D) indicates the histogram of DAT mRNA density. Data were indicated as the mean ± SEM (*n* = 20 mice/group). (a) Saline group, (b) IDPN group, (c) IDPN+Tia group, and (d) IDPN+JPZDD group. DAT protein and its mRNA were tagged with black arrow. * indicates *P* < 0.05 and ** indicates *P* < 0.01 compared with IDPN group, respectively; ^##^ indicates *P* < 0.01 compared with saline group; ▲ indicates *P* < 0.05 and ▲▲ indicates *P* < 0.01 compared with IDPN+Tia group, respectively.

In order to further investigate the activity and quantity of DAT in the striatum under diverse medications, DAT protein and mRNA expression were assessed by immunohistochemistry and in situ hybrization. DAT protein was decreased in IDPN group compared with that in saline group (*P* < 0.05) (Figures 3(a)-3(b)). Moreover, IDPN+JPZDD and IDPN+Tia, respectively, could prompt the abundance of DAT, and more notable improvement was observed in JPZDD treated mice (*P* < 0.05). The level of DAT mRNA (Figures 3(c)-3(d)) also revealed that IDPN alone dramatically reduced DAT mRNA abundance. However, treatments of JPZDD and Tia, respectively, increased DAT mRNA abundance by 18% and 41% as compared to that of IDPN group.

## 4. Discussion

From the viewpoint of TCM, children with TS usually suffer from excessive energy of the liver and deficiency of the spleen. Based on this theory we hypothesized that imbalance of liver and spleen in TS patients generates endogenous liver wind and spleen phlegm, where liver wind agitates, and spleen phlegm obstructs the channels. According to this hypothesis, we created a recipe named as JPZDD that includes two ancient formulae of Liu-Jun-Zi-Tang (LJZT) and Xie-Qing-Wan (XQW). Its characteristic for treating TS is particularly by strengthening the spleen, supplemented by inhibiting the liver. The herbs in JPZDD were strictly based on the compatibility theory of TCM. *Pseudostellaria heterophylla* Pax, *Poria cocos* Wolf, and *Atractylodes macrocephala* Koidz could invigorate the spleen-qi. *Citrus reticulata* Blanco and *Pinellia ternata* Breit could eliminate the phlegm. *Gentiana scabra* Bge, *Saposhnikovia divaricata* Schischk, and *Uncaria rhynchopylla* Jacks could clear away the liver fire and calm down the liver wind. *Ligusticum chuanxiong* Hort and *Angelica sinensis* Diels could nourish the liver blood and harmonize the live wind.

Evidence based on the neurocognitive literature earlier indicated that diminished executive functions (EFs) such as working memory, cognitive flexibility are present in TS children [13, 14]. Neurotoxic species catalyzed by monoamine oxidases (MAOs) could induce mitochondrial damage and neuronal apoptosis in the prefrontal cortex and basal ganglia, the cascade of which is assumed to contribute to tics and hyperactive behavior [15, 16]. Combining with the existing studies, efficiencies of the active compounds (Table 1) in JPZDD are complicated but relatively unified. Polysaccharides, total alkaloids, and tetramethylpyrazine have been proven to ameliorate the cerebral hypoxic ischemia and restore the balance of oxidants and reductants in the cortex [17–19]. Sodium ferulate has potent protective effects against glutamate-induced neurotoxicity on developing mouse fetal brain [20]. Rhynchophylline protects the neuronal cultures against MA exposure and promptly attenuate intracellular calcium overload triggered by MA challenge [21]. Gentianine and senkyunolide increase the inhibitory transmitters which antagonize the excessive excitability in the brain regions [22, 23]. In brief, its anti-tics effect might partly attribute to the synergistic interactions consisting of antioxidation and cognitive improvement.

Previous studies in our group have been focused on JPZDD regulation of monoamine neurotransmitters and their metabolites in TS mice together with its efficacy on anti-tics. The findings suggested that 10 g kg^−1^ JPZDD (equivalence of 5-fold dosage for children) could decrease the levels of DA, DOPAC, HVA, 5-HIAA, and NE in the striatum and peripheral blood. It is tempting to discover that XQW is the predominant inhibitor acting on DA efflux. Detected by high performance liquid chromatography (HPLC), the level of DA in IDPN+XQW group is lower than the other four groups, and results of the falling percentage are 18.54% (compared with IDPN group), 21.66% (compared with IDPN+Tia group), 13.02% (compared with IDPN+JPZDD group), and 21.21% (compared with IDPN+LJZT group), respectively. Further research about the anti-tics effect of JPZDD on TS mice model has not been carried out, though it has been testified that JPZDD and XQW could reduce the times of spontaneous hyperkinesias [24, 25]. Therefore, 10-fold dosage was given in this study, and the satisfactory results were achieved. Overly rigid sequences of action were ameliorated apparently shown from the chart depicting the severity of stereotype scored at each time points. The broken line emerged as a stable downtrend in IDPN+JPZDD group, whereas a small bounce during the 3rd and 4th week in IDPN+Tia group then declined. The variability exhibited is more or less in agreement with that in clinic which reflected a long-term curative predominance of JPZDD.

Through abnormal expression of DAT exists in TS patients, it is still unclear what role it plays in formation of the disease. A much greater density of DAT may reflect the preponderance of DA innervation in together with vesicular monoamine transporter type 2, but few pros put forward which count this change as an offset to thrust of hyperdopaminergia. An animal experiment has confirmed that DAT knockdown mutant mice cause extracellular DA levels in the neostriatum to rise to 170% of wild-type control levels, and sequential super stereotypy occurs in these complex fixed action patterns of hyperdopaminergic mutant mice [26].

Hypothesis of supersensitive postsynaptic DA receptor (DR) is a complementary standpoint supported by the typical evidence from clinical response of TS patients to DR antagonists such as haloperidol. An intriguing phenomenon arose that reduced cerebral spinal fluid baseline and turnover levels of homovanillic acid in untreated cases recovered to normal range after drugs. By antagonizing the sites on postsynaptic DR, the binding of DR will be reduced. Early opinions suggested that chronic haloperidol treatment had a higher impact on DR than DAT, which brought about increased DRD2 specific binding but lack of an adaptative change of DAT [27]. In a subsequent literature, we found that DRD2 could interfere with the expression and reuptake capacity of DAT by virtue of DRD2-DAT interaction, which further influenced the clearance of DA. According to this perspective, exogenous and endogenous factors may become determinants to concentration of DA in synaptic cleft secondary to reaction of momentary variation of DAT.

3,3′-iminodipropionitrile (IDPN) as a neurotoxin could produce the rodents a persistent behavior syndrome resembling TS. The IDPN-induced dyskinesia was considered due to activation of DA neurons and enhanced by administration of L-dopa, which increases DA concentration. Moreover, hyperfunction of DA neuronal system could be relieved by Tia [28]. 

Tia, acting as a selective D2R antagonist, has a supersensitive affinity for D2R in the ventral striatum and parts of the limbic system. It could antagonize the dyskinetic orolinguofacial symptoms triggered by injections of DA in the striatum which is assumed to inhibit the stimulation of DA2 neurons that cause dyskinetic reactions. A randomized, double-blind, placebo-controlled crossover study involving 17 TS children testified its significant reduction of tic symptomatology [29]. The positive anti-tics profile with doses has also been proven in rodents [30], otherwise high doses (ED_50_ = 60 mg kg^−1^, i.p.) of Tia were found to decrease spontaneous locomotor activity induced by apomorphine 3 times higher than haloperidol [31].

Hence, in this study, IDPN and Tia were chosen as inducer and control drug in comparison with JPZDD, respectively. In line with the existing reports, the induced mice demonstrated a series of stereotyped behaviors mainly displayed as lateral and vertical head twitching, circling, sniffing, and grooming, and these symptoms lasted at least 2 months without medicine intervention. The impairment of DAT in IDPN group worsened the most sharply in four groups. JPZDD and Tia could increase the expression of DAT protein and mRNA in varying degrees. 

**Figure 4:**
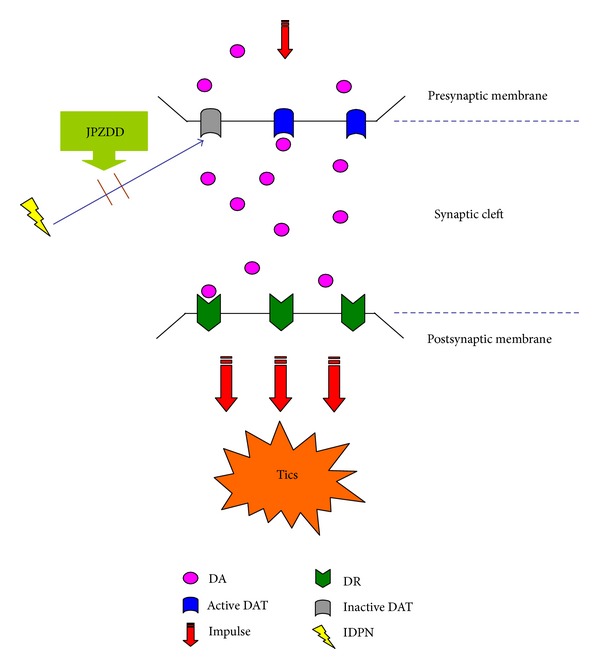
A schematic plot for IDPN-induced tics in DA neurons of the striatum. The number of active DAT decreases triggered by IDPN, which leads to a mass of DA staying in the synaptic cleft. A higher concentration of DA may prolong the time span of DA-DR interconnection, facilitate excessive excitatory signals which may initiate abnormal motors such as tics.

Simultaneously, the affinity binding to radioligand tested by neuroimaging indicated JPZDD and Tia might improve the bioactivity of DAT. Combined with previous results, it is reasonable to postulate that downregulation of DAT in striatum which leads directly to higher concentration of DA is one potential pathogenesis of TS (Figure 4), and the anti-tics effect of JPZDD may be connected with upregulation of DAT expression. 

Thus far, a typical drug for patients with TS targeting at DAT has not been on market due to lack of convincing clinical evidences [32]. Currently, haloperidol and sulpiride as the representatives of first-line antipsychotics are widely accepted for their effectiveness in clinic. However, their adverse reactions such as extrapyramidal symptoms, tardive dyskinesia, drowsiness, and hyperprolactinemia usually daunt TS patients [33]. The creation of JPZDD largely avoided the side effects above [9]. 

Some limitations existed in the course of the experiment. Firstly, on account of smaller striatum in brain tissue, we just obtain the sample as a whole where the pathological damages are hardly located precisely. Secondly, respecting a limited number of TS mice in each group, it is difficult to relate the injury severity with the degrees of behavioral scores, which could be used as a reference for judging the severity and prognosis in clinic. Thirdly, this study did not explain the phenomenon of higher expression of DAT in some TS patients.

In conclusion, JD is an effective formula for treating TS on basis of upregulating the DAT expression in the striatum, which may indirectly reinforces the recapture of DA back to the presynaptic membrane. Findings of this study also provide a new recognition that the expression of DAT in DA system is an important factor contributing to occurrence and development of TS. 

## Figures and Tables

**Table 1:** Composition and active compounds of JPZDD.

Components	Voucher specimens number	Part used	Active compounds	Amount used (g)
*Pseudostellaria heterophylla* (Miq.) Pax ex Pax et Hoffm	120301	Root	Polysaccharides	10
*Atractylodes macrocephala* Koidz	120502	Rhizoma	Atractylenolide III	10
*Poria cocos* (Schw.) Wolf	120403	Sclerotium	Polysaccharides	10
*Pinellia ternata* (Thunb.) Breit	120201	Tuber	Total alkaloids	6
*Citrus reticulata* Blanco	120202	Mature pericarp	Hesperidin, citrus flavonoids	6
*Saposhnikovia divaricate* (Turez.) Schischk	120401	Root	Polysaccharides, chromones	6
*Gentiana scabra* Bge	120501	Root	Gentianine	3
*Angelica sinensis* (Oliv.) Diels	120402	Root	Z-Ligustilide, sodium ferulate	10
*Ligusticum chuanxiong* Hort	120501	Root	Senkyunolide, tetramethylpyrazine	6
*Uncaria rhynchopylla* (Miq.) Jacks	111101	Stem with hooks	Rhynchophylline	10

**Table 2:** Behavior measurements referring to evaluating grades of stereotypy.

Score	Stereotypy
0	No stereotypy or normal activity
1	Discontinuous circling behavior (clockwise/counterclockwise circling)
	Occasional head twitching
2	Occasionally vertical dyskinetic head and neck movements
	Occasional sniffing, licking, and biting
3	Continuous circling behavior, increased body raising
	Increased sniffing, repetitive grooming (such as paw-to-mouth movements)
4	Increased lateral and vertical dyskinetic head and neck movements
